# Acute Respiratory Distress Syndrome Secondary to Influenza A Infection in a Patient With No Significant Risk Factors: A Case Report

**DOI:** 10.7759/cureus.87803

**Published:** 2025-07-13

**Authors:** Alain Khouri, Mariana Helou, Ahmad Haj Hussein, Alain Tanios, Ramzi Nakhle

**Affiliations:** 1 Emergency Department, Lebanese American University School of Medicine, Beirut, LBN

**Keywords:** acute hypoxemic respiratory failure, acute respiratory distress syndrome [ards], methicillin resistant staphylococcus aureus (mrsa), pneumonia, type a influenza infection

## Abstract

Influenza A can present with a range of respiratory symptoms and, in some cases, lead to serious complications. One such complication is acute respiratory distress syndrome (ARDS), which may result from an intense immune response to viral replication in the lungs.

We describe a 52-year-old woman with no known comorbidities who developed rapidly progressive ARDS due to influenza A infection. Her clinical course was further complicated by a superimposed methicillin-resistant *Staphylococcus aureus* (MRSA) pneumonia. She required admission to the intensive care unit (ICU), intubation, and mechanical ventilation.

This case illustrates that influenza A can lead to severe respiratory failure even in individuals without underlying health conditions. It underscores the importance of early recognition of complications and timely escalation of care in patients with influenza-like illness.

## Introduction

Influenza is a common viral infection that primarily affects the respiratory system. The virus replicates in the respiratory epithelium and can lead to severe lung inflammation and respiratory distress when the viral load is high and the host immune response is robust [1]. This may progress to acute ARDS.

Acute respiratory distress syndrome (ARDS) is an acute inflammatory lung injury characterized by increased pulmonary vascular permeability, bilateral alveolar infiltrates, and hypoxemia [2]. Inflammatory involvement can extend systemically and result in multiorgan failure. In influenza infection, ARDS develops due to widespread involvement of the lower airways either by direct viral inoculation or through immune-mediated damage [1].

Classical symptoms of influenza infection include fever, chills, myalgias, and cough. Complications such as secondary bacterial pneumonia, most commonly caused by *Staphylococcus aureus* (*S. aureus*) or *Streptococcus pneumoniae *(*S. pneumoniae*), and ARDS may occur, especially in immunocompromised patients or those with chronic lung disease [3]. However, previously healthy individuals can also develop severe respiratory failure, as observed during the 2009 H1N1 pandemic [1].

We report the case of a 52-year-old woman who presented with severe, rapidly progressing ARDS secondary to influenza A virus infection, despite having no significant comorbidities.

## Case presentation

A 52-year-old woman with a medical history of dyslipidemia, migraines, and a prior minor traumatic intracranial bleed (treated conservatively two years prior with no sequelae) presented to the emergency department (ED) with severe, sudden-onset dyspnea, chest discomfort, and a severe sore throat described as a burning sensation, associated with odynophagia. She also reported rhinorrhea, productive cough, a milder sore throat, and chills for one week prior to presentation, without documented fever.

**Figure 1:**
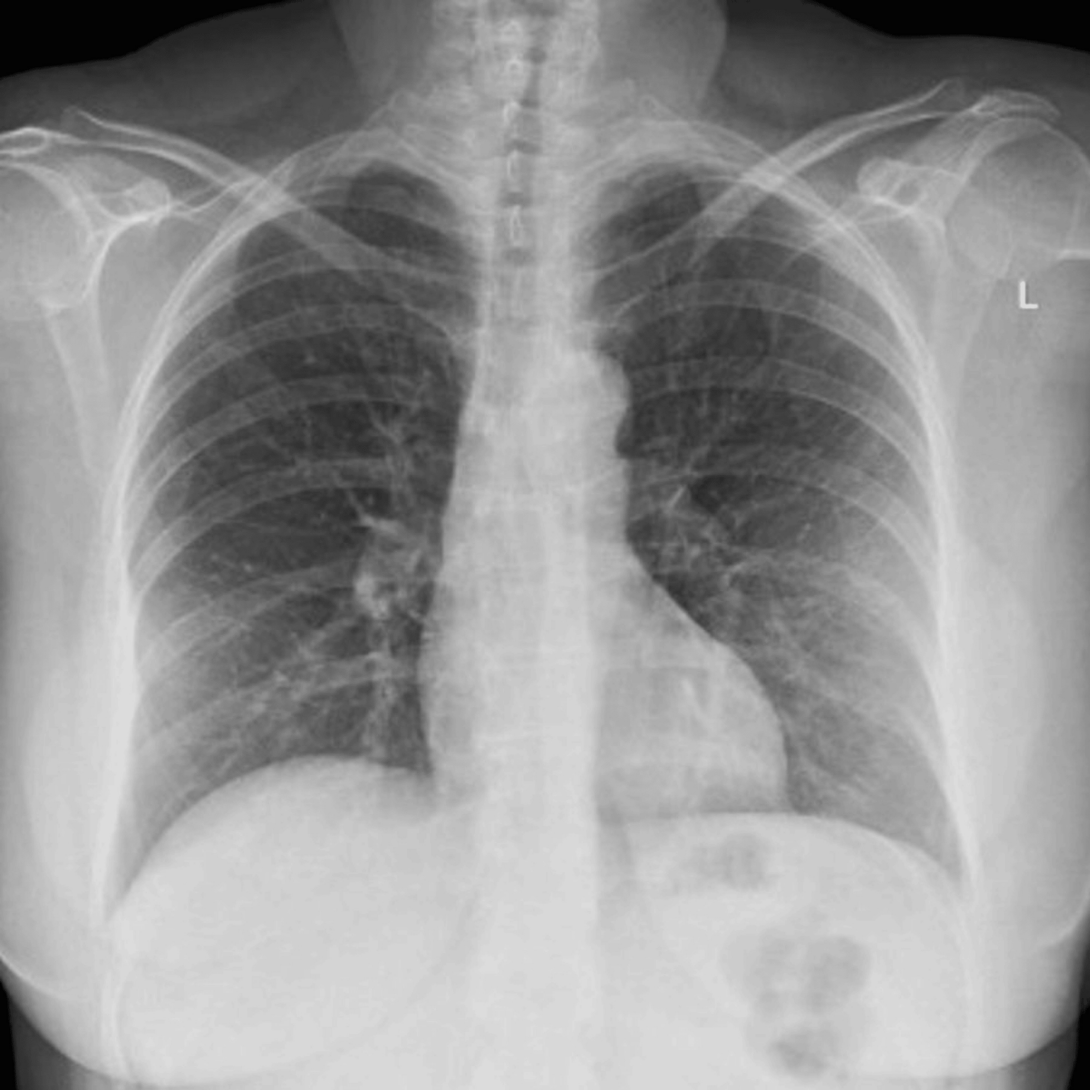
Chest X-ray one day prior to admission.

She had presented to the ED one day earlier for worsening sore throat and persistent symptoms. A chest X-ray (Figure 1) at that time revealed increased bronchial markings and faint, hazy, ill-defined infiltrates in the periphery of both lower lung fields, with no evidence of consolidation. Her inflammatory markers were mildly elevated, WBC = 8.20, CRP = 33 (Table 1), and a rapid antigen nasopharyngeal swab was positive for influenza A. She was clinically stable with normal vital signs and discharged with symptomatic treatment and oseltamivir.

**Table 1:** Laboratory results WBC: white blood cells; CRP: C-reactive protein

Tests	1-Results	2-Results	Reference Values
WBC	8.2	3.33	4.0 - 10 x 10^9/L
CRP	33	283.9	< 5mg/L

Upon return to the ED one day after the initial ED presentation, the patient was tachypneic and tachycardic (HR = 112 bpm, sinus rhythm on ECG), unable to lie flat, and had an SpO₂ of 87% on room air. Blood pressure was 130/83 mmHg, and her temperature was 37.2°C. Arterial blood gases (ABG) showed mild respiratory alkalosis (pH = 7.47, pCO₂ = 31 mmHg) and hypoxia (PaO₂ = 50 mmHg, SpO₂ = 88% on room air). Her oxygen saturation continued to decline, requiring escalation of oxygen therapy. She was stabilized with a SpO₂ of 95% on 60 L/min high-flow nasal cannula (HFNC) within one hour. She experienced a transient hypotensive episode, responsive to fluid resuscitation, and had a low-grade fever (38°C).

**Figure 2:**
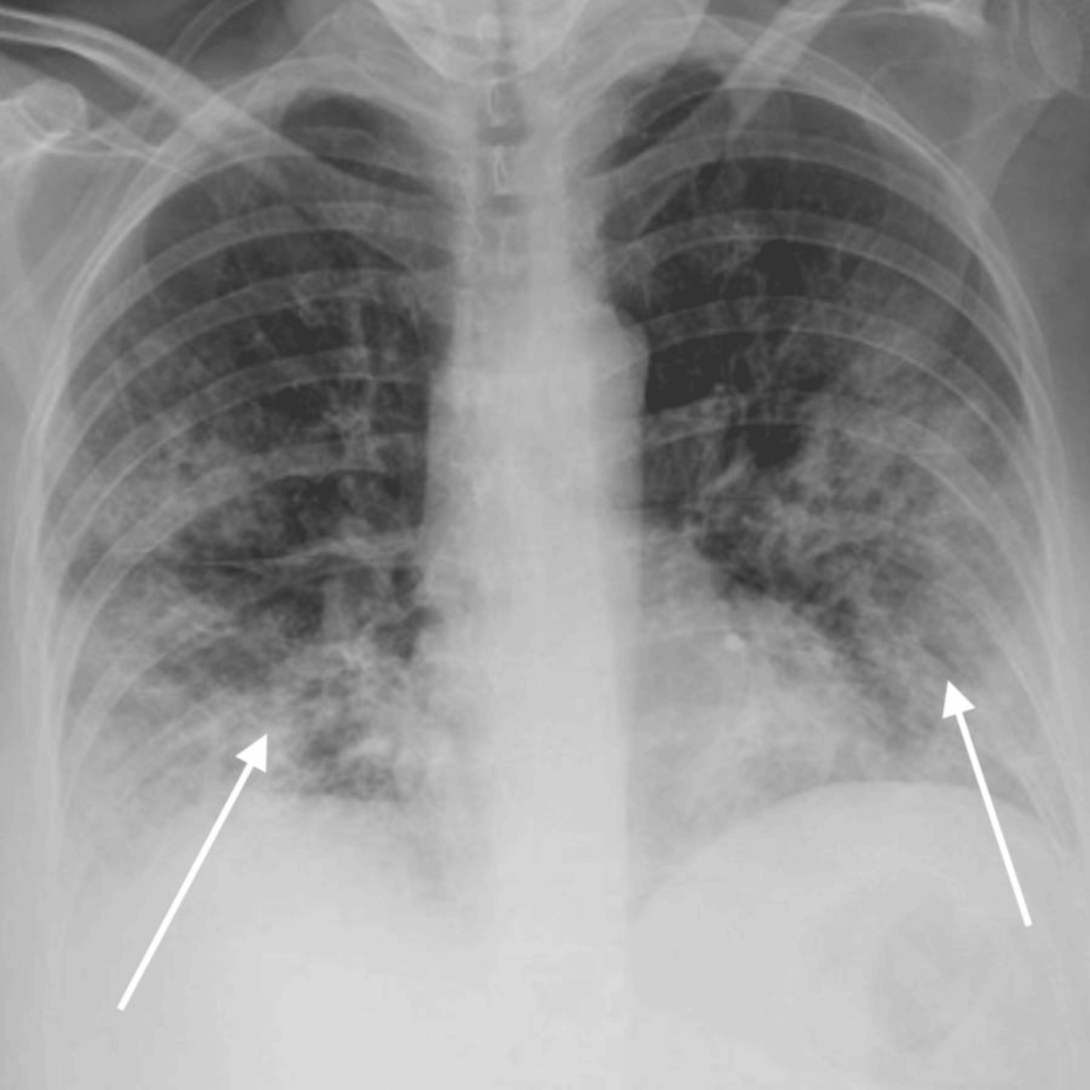
Chest X-ray on admission

**Figure 3:**
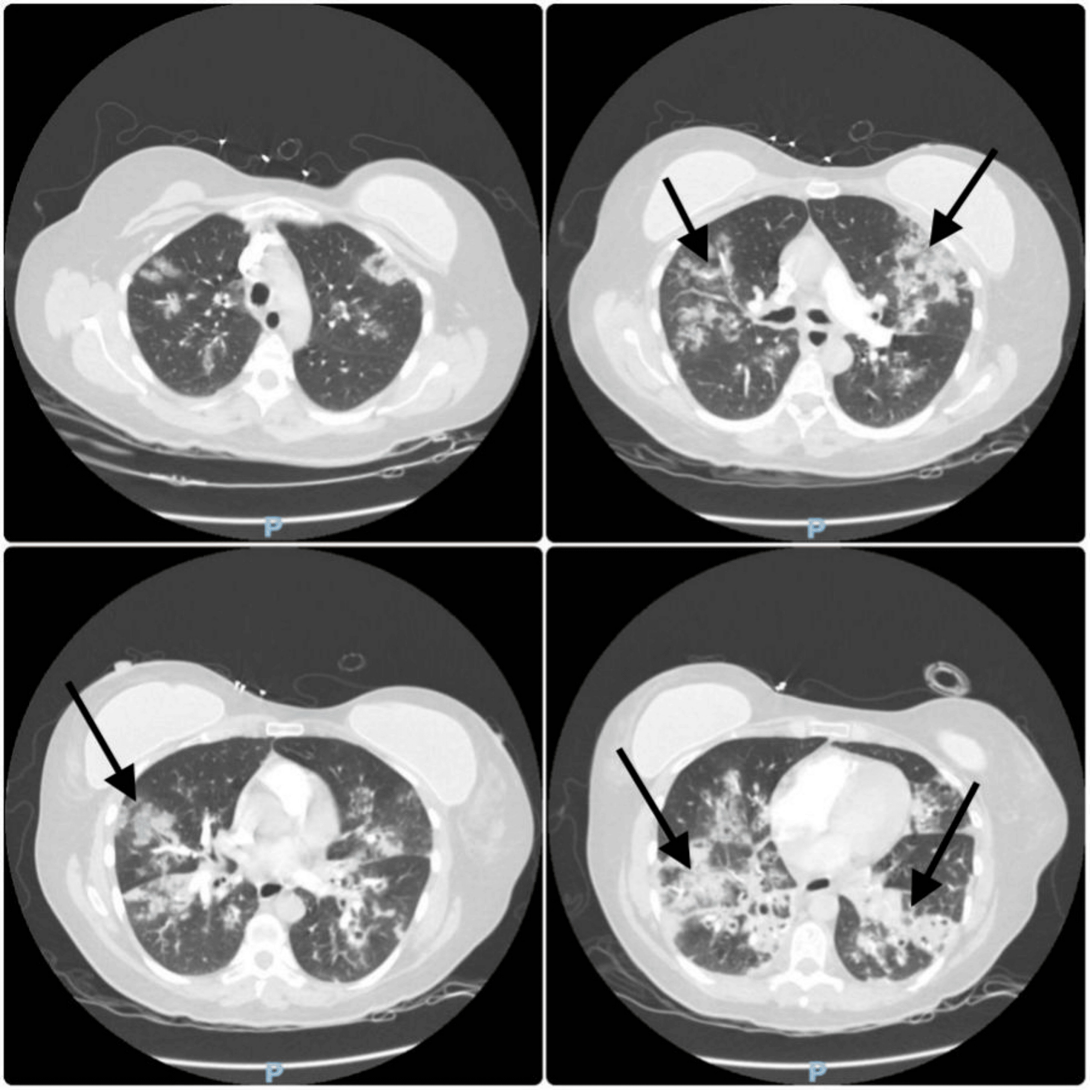
CT chest on admission CT: computed tomography

Repeat ABG on HFNC showed resolution of respiratory alkalosis and improved oxygenation (PaO₂ = 73 mmHg, SpO₂ = 95%). Auscultation revealed good bilateral air entry with diffuse rhonchi. Repeat chest X-ray and CT scan demonstrated bilateral patchy infiltrates (Figures 2-3), consistent with ARDS, with concern for a superimposed bacterial infection. Laboratory results showed a marked increase in CRP to 283.9 and a decline in WBC to 3.33 (Table 1). Given her PaO₂/FiO₂ ratio of 73 (on 100% FiO₂ at 60 L/min), she met the diagnostic criteria for ARDS under the updated global definition.

**Figure 4:**
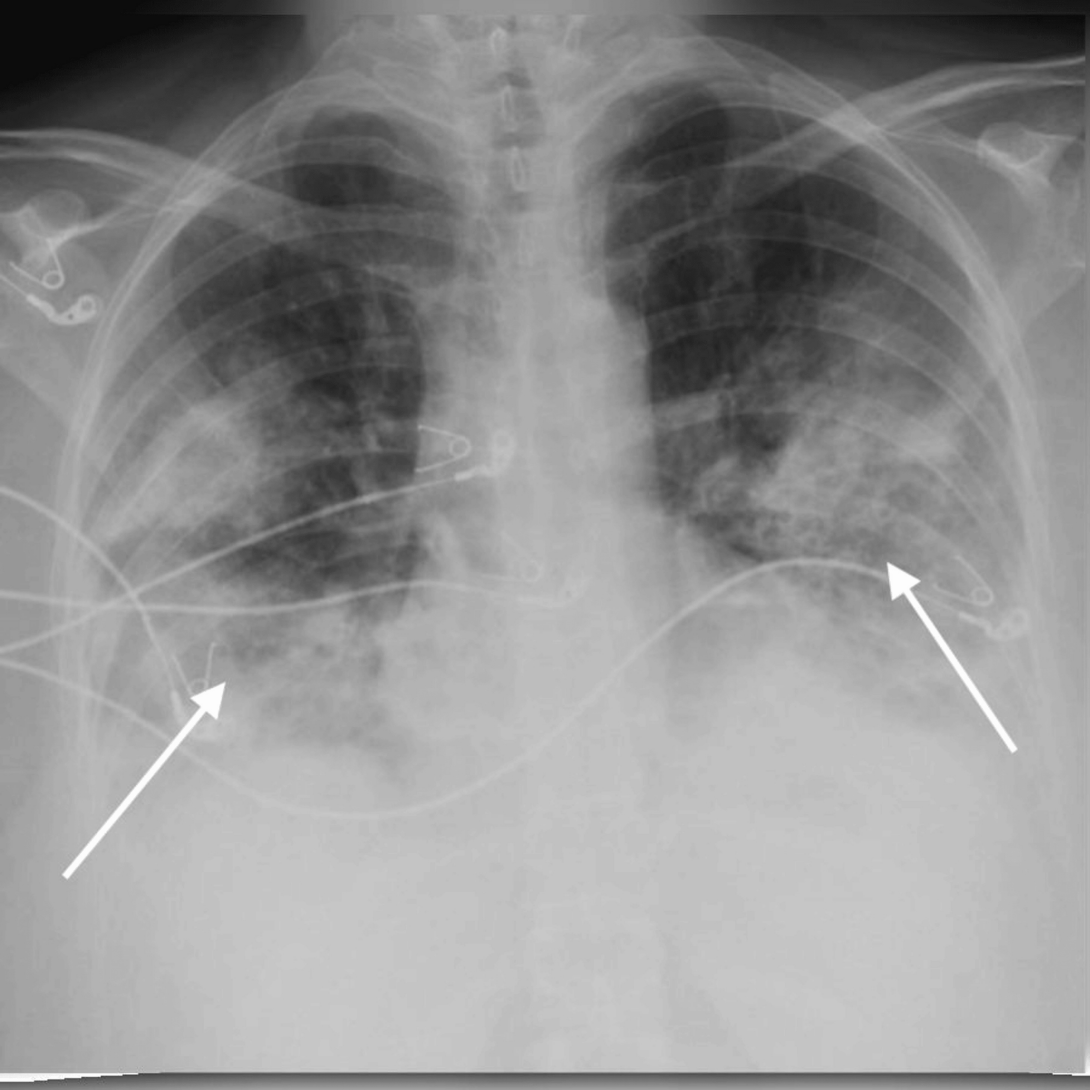
Chest X-ray 24 hours after admission

Infectious disease consultation was obtained, and broad-spectrum antibiotics (ceftriaxone, levofloxacin, and teicoplanin) were initiated. Oseltamivir was continued. The patient was admitted to the ICU for monitoring and treatment. Over the following 24 hours, her condition deteriorated, her chest X-ray worsened (Figure 4), and she required intubation and mechanical ventilation.

Sputum culture on admission was positive for superimposed methicillin-resistant* S. aureus *(MRSA); other cultures remained negative. One month after the initial presentation, she remains in the ICU. She is conscious and oriented, with a tracheostomy tube in place, and is still on high positive end-expiratory pressure (PEEP) and FiO₂ on pressure support ventilation (PSV).

## Discussion

This case highlights the rapid progression of ARDS secondary to influenza A in a previously healthy 52-year-old woman, an uncommon but documented outcome during certain influenza outbreaks, particularly H1N1 [1].

The influenza virus typically infects the respiratory epithelium from the upper airway down to the alveoli [1,2]. While most infections are limited to the upper respiratory tract, influenza can cause severe complications, including viral pneumonia and ARDS, particularly in high-risk individuals [4]. Risk factors include age extremes (<5 or >65 years), smoking, immunosuppression, and chronic cardiopulmonary disease [1]. This case, however, emphasizes that even patients without these risk factors may develop severe disease.

ARDS results from widespread pulmonary inflammation marked by apoptosis, necrosis, and increased alveolar-capillary permeability, leading to alveolar edema and impaired gas exchange [5]. Common triggers include pulmonary infections and extrapulmonary insults such as sepsis, trauma, massive transfusion, pancreatitis, drug overdose, and inhalation of toxic fumes [5,6].

In influenza-associated ARDS, high viral load and an exaggerated immune response contribute to lung injury and alveolar remodeling [1]. This case met the new global ARDS definition, which includes patients on HFNC receiving ≥30 L/min and a PaO₂/FiO₂ ratio <300 [6].

Secondary bacterial pneumonia is another frequent complication of influenza, particularly due to* S. pneumoniae *or* S. aureus*, including MRSA [4]. Unlike COVID-19, secondary bacterial infections are more common in hospitalized patients with influenza [4]. In our patient, MRSA was identified on sputum culture, likely exacerbating the ARDS progression.

Despite the early initiation of oseltamivir and empiric broad-spectrum antibiotics, the patient’s condition worsened, requiring mechanical ventilation. Early antiviral treatment has been associated with reduced mechanical ventilation duration and ICU stay [7]. Nevertheless, this case illustrates how influenza can rapidly progress despite timely interventions.

ARDS mortality has declined over the past two decades, now ranging between 9-20%, though it remains higher in older adults [5]. Compared to COVID-19-related ARDS, patients with influenza A exhibit more severe hypoxemia but similar mortality rates [8]. Death typically results from septic shock or multiorgan failure. Survivors often experience long-term sequelae including dyspnea, reduced exercise tolerance, and cognitive impairment [5].

## Conclusions

Influenza A virus causes a broad spectrum of illness, ranging from mild respiratory symptoms to life-threatening ARDS. Clinicians should educate high-risk populations about influenza symptoms and the importance of early testing and treatment. Seasonal influenza vaccination remains a critical preventive measure. This case also serves as a reminder that severe complications such as ARDS can develop in previously healthy individuals, emphasizing the need for high clinical vigilance.

## References

[1] Kalil AC, Thomas PG (2019). Influenza virus-related critical illness: pathophysiology and epidemiology. Crit Care.

[2] Gacouin A, Lesouhaitier M, Reizine F (2020). Short-term survival of acute respiratory distress syndrome patients due to influenza virus infection alone: a cohort study. ERJ Open Res.

[3] Bal A, Casalegno JS, Melenotte C (2020). Influenza-induced acute respiratory distress syndrome during the 2010-2016 seasons: bacterial co-infections and outcomes by virus type and subtype. Clin Microbiol Infect.

[4] Uyeki TM, Hui DS, Zambon M, Wentworth DE, Monto AS (2022). Influenza. Lancet.

[5] Diamond M, Peniston HL, Sanghavi DK, Mahapatra S (2024). Acute respiratory distress syndrome. StatPearls.

[6] Matthay MA, Arabi Y, Arroliga AC (2024). A new global definition of acute respiratory distress syndrome. Am J Respir Crit Care Med.

[7] Moreno G, Rodríguez A, Sole-Violán J (2021). Early oseltamivir treatment improves survival in critically ill patients with influenza pneumonia. ERJ Open Res.

[8] Krynytska I, Marushchak M, Birchenko I, Dovgalyuk A, Tokarskyy O (2021). COVID-19-associated acute respiratory distress syndrome versus classical acute respiratory distress syndrome (a narrative review). Iran J Microbiol.

